# Eculizumab treatment: stochastic occurrence of C3 binding to individual PNH erythrocytes

**DOI:** 10.1186/s13045-017-0496-x

**Published:** 2017-06-19

**Authors:** Michela Sica, Tommaso Rondelli, Patrizia Ricci, Maria De Angioletti, Antonio M. Risitano, Rosario Notaro

**Affiliations:** 1 Laboratory of Cancer Genetics and Gene Transfer, Core Research Laboratory – Istituto Toscano Tumori (CRL-ITT), AOU Careggi, viale Pieraccini 6, 50139 Florence, Italy; 2 Hematology, Department of Clinical Medicine and Surgery, Federico II University, Naples, Italy; 3 ICCOM-CNR, Sesto Fiorentino, Florence Italy

**Keywords:** Paroxysmal nocturnal hemoglobinuria, Complement blockade, Intravascular hemolysis, Extravascular hemolysis, Complement C3, Complement C5

## Abstract

**Background:**

C5 blockade by eculizumab prevents complement-mediated intravascular hemolysis in paroxysmal nocturnal hemoglobinuria (PNH). However, C3-bound PNH red blood cells (RBCs), arising in almost all treated patients, may undergo extravascular hemolysis reducing clinical benefits. Despite the uniform deficiency of CD55 and of CD59, there are always two distinct populations of PNH RBCs, with (C3+) and without (C3−) C3 binding.

**Methods:**

To investigate this paradox, the phenomenon has been modeled in vitro by incubating RBCs from eculizumab untreated PNH patients with compatible sera containing eculizumab, and by assessing the C3 binding after activation of complement alternative pathway.

**Results:**

When RBCs from untreated patients were exposed in vitro to activated complement in the context of C5-blockade, there was the prompt appearance of a distinct C3+ PNH RBC population whose size increased with time and also with the rate of complement activation. Eventually, all PNH RBCs become C3+ to the same extent, without differences between old and young (reticulocytes) PNH RBCs.

**Conclusions:**

This study indicates that the distinct (C3+ and C3−) PNH RBC populations are not intrinsically different; rather, they result from a stochastic *all-or-nothing* phenomenon linked to the time-dependent cumulative probability of each individual PNH red cell to be exposed to levels of complement activation able to trigger C3 binding. These findings may envision novel approaches to reduce C3 opsonization and the subsequent extravascular hemolysis in PNH patients on eculizumab.

**Electronic supplementary material:**

The online version of this article (doi:10.1186/s13045-017-0496-x) contains supplementary material, which is available to authorized users.

## Background

Paroxysmal nocturnal hemoglobinuria (PNH) is an acquired disorder of hematopoiesis characterized by a somatic mutation in the *PIGA* gene that prevents or impairs the synthesis of glycosylphosphatidylinositol (GPI) anchors [1, 2]. The deficiency on red blood cells (RBCs) of GPI-anchored proteins [3, 4], including the complement regulators CD55 [5, 6] and CD59 [7], results in chronic intravascular hemolysis with recurrent exacerbations, anemia, smooth muscle cell dystonia, and high risk of thrombosis [4, 8–10].

The blockade of terminal complement pathway by eculizumab [11], a monoclonal antibody (moAb) against complement component 5 (C5), abrogates intravascular hemolysis with the consequent normalization of lactate dehydrogenase (LDH) levels in almost all patients suffering from PNH. This treatment has proven to be safe and clinically effective in hemolytic PNH patients [12–14], except those in which bone marrow failure is the major cause of anemia [15, 16].

The persistence (or the recurrence) of intravascular hemolysis is observed only in few conditions: (i) Japanese patients carrying a rare polymorphism of C5 [17], (ii) patients with an increased eculizumab turnover requiring extra-dosage (pharmacokinetic breakthrough) [18], and (iii) patients who occasionally experience transient episodes of intravascular hemolysis because of massive complement activation during infections or inflammatory disorders (pharmacodynamic breakthrough) [18–21]. Despite these small and infrequent limits, the treatment with eculizumab has radically changed the natural history of PNH since in most patients it reduces anemia [12, 13] and thrombosis [22], and improves quality of life and survival [14].

However, the abrogation of intravascular hemolysis is not the only relevant change in PNH pathophysiology associated with eculizumab treatment. In fact, at variance with PNH patients not treated with eculizumab, a population of GPI-negative (PNH) RBCs bound with fragment of complement component 3 (C3) appears in almost all patients on eculizumab [23] and, in some patients, also the less-sensitive direct antiglobulin test may turn positive [24]. The PNH RBCs bound with C3 become apparent because PNH RBCs, spared from hemolysis by the blockade of the terminal complement cascade, remain unable to control the early steps of the ongoing complement activation. Eventually, these PNH RBCs, once opsonized with complement, become potential targets of phagocytosis by macrophages, with consequent variable degrees of extravascular hemolysis [23]. Accordingly, in PNH patients on eculizumab, the extent of C3 binding correlates with reticulocyte count, the in vivo half-life of ^51^Cr-labeled RBCs is reduced and there is an excess of spleen and liver ^51^Cr uptake [23]. This extravascular hemolysis is clinically relevant because it can limit the efficacy of eculizumab to the point that some patients may remain transfusion-dependent [14, 22–25]. The heterogeneity of mechanisms controlling C3 binding and/or removal of C3+ PNH RBCs are likely to account for the variable extent of C3 binding and of the consequent extravascular hemolysis. Part of this variability may result from genetic diversity in genes coding for endogenous regulators of complement: indeed, we have recently shown that polymorphisms of complement receptor 1 (*CR1*) gene are associated with the transfusion need of patients on eculizumab [26].

The most intriguing biological feature of C3 binding is that two distinct populations of PNH RBCs are always present in patients on eculizumab, one with (C3+) and one without (C3−) C3 binding, despite the uniform deficiency of the GPI-anchored proteins on all PNH RBCs [23]. The understanding of this phenomenon is of more than academic importance because, depending on the answer, one might explore different ways to overcome the consequent clinical problems. In this paper, by reproducing in vitro C3 binding in the presence of C5 blockade, we provide evidence suggesting a stochastic model for the emergence of these two distinct, C3+ and C3−, populations of PNH RBC.

## Methods

### Patients and samples

Peripheral blood samples were collected from 31 healthy donors and from 31 patients with frank hemolytic PNH. Nine of these patients were on eculizumab, 13 were not and 9 have been studied before and on eculizumab. Signed informed consent was obtained according to an IRB-approved protocol. RBCs and sera were promptly separated from freshly collected samples. RBCs were washed three times in saline just before use. Sera were stored at −80 °C.

### In vitro complement activation

The approach we have used derives from the diagnostic Ham test traditionally used to test the susceptibility of PNH RBCs to complement-mediated lysis by activation of the complement alternative pathway (CAP) [27–29]. Briefly, 2% RBC suspensions were incubated at 37 °C in saline with 84% of pooled ABO-compatible sera from either healthy donors (normal) or from PNH patients on eculizumab (with eculizumab). Sera with eculizumab were collected 1 h after infusion from PNH patients stably treated with eculizumab (900 mg each 2 weeks) and with normal LDH during the last 3 months (in these samples the expected concentration of eculizumab is between 200 and 500 μg/ml: [12, 30] a concentration that is 5–14 times higher than the 35 μg/ml reported as the minimal effective level of eculizumab [12]. In selected experiments, eculizumab, obtained from remnants in the infusion lines, was added (400 μg/ml).

CAP was activated by either spontaneous activation (incubation in sealed tubes with 100% atmospheric air) [31] or by mild acidification (HCl 0.016 M: the final pH was between 6.8 and 6.5) [31–34]. All the procedures were performed in sterile conditions. The hemolysis of normal RBCs has been quantified by a direct spectrophotometric method [35]. In both experimental models, the lysis of RBCs from healthy donors was less than 2%. The hemolysis of PNH RBCs has been quantified by comparing the relative variation of the percentage of normal RBCs (GPI-positive resistant to complement) with that of PNH RBCs (GPI-negative sensitive to complement) [36].

### Flow cytometry

C3 binding was assessed with anti-C3d-neoantigen (A250, Quidel, USA) secondary stained with polyclonal rabbit-anti-mouse antibodies (Dako Cytomation, Denmark). In selected experiments, RBCs were stained also with anti-C3b (H11, Serotec), anti-iC3b (A209, Quidel), anti-C3c (MCA2605, Serotec), anti-Bb (A227, Quidel), and the proper secondary polyclonal rabbit-anti-mouse antibodies (Dako Cytomation). PNH RBCs have been identified with anti-CD59 (Mem43, Serotec, UK). Intact and lysed (ghost) RBCs have been identified by physical parameters as previously described [37]; in selected experiments, the identity of intact and of ghost RBCs has been confirmed by staining with anti-Glycophorin A moAb (GA-R2, BD Becton Dickinson, NJ, USA).

Reticulocytes have been stained with the polymethine dye (Ret Search II, Sysmex) that, upon excitation at 633 nm, emits between 650 and 670 nm. Briefly, 1 volume of RBC suspension was mixed in 4 volumes of “Ret Search” dilution buffer (Sysmex) and stained by adding the polymethine dye just before flow cytometry analysis [38].

Flow cytometry has been performed with either FACSCanto (BD) or Accuri C6 (BD). Fluorescent-activated cell sorting (FACS) has been performed with FACSAria III (BD).

### Statistical analysis

Statistical analysis was performed using non-parametric tests: Wilcoxon signed paired and Friedman rank sum tests as suitable. Statistical significance was accepted for any *P <* 0.05.

## Results

### Analysis of C3 fragments bound to PNH red blood cells in patients on eculizumab

**Fig. 1:**
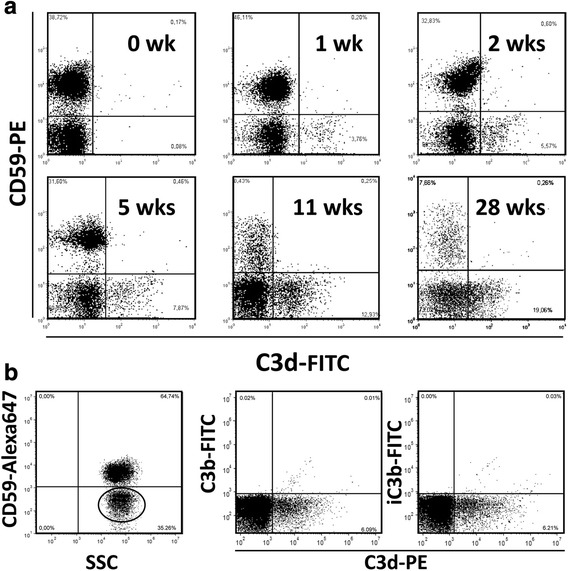
In vivo C3 binding on red cells of PNH patients on eculizumab. **a** Kinetics of C3 binding on red cells from a representative PNH patient during eculizumab treatment. Before treatment (0 week), no red cell binds C3, whereas starting from 1 week of treatment three populations of red cells are displayed: one population of normal red cells (CD59 + C3−) and two distinct populations of PNH (CD59-negative) red cells, one with (C3+) and one without (C3−) fragments bound on their surface. **b** Characterization of C3 fragment bound to erythrocytes from PNH patient on eculizumab. The *left panel* shows the normal (CD59-positive) and PNH (CD59-negative) populations (gated by the *elliptic mark*). The PNH (CD59-negative) population (see gate in the *left panel*) has been analyzed with anti-C3d and either anti-C3b (*middle panel*) or anti-iC3b (*left panel*). SSC: side scatter

In most of the PNH patients undergoing eculizumab treatment, there is a relative and an absolute increase of PNH RBCs because complement blockade prevents their complement-mediated lysis. In parallel, the percentage of PNH RBCs with C3 binding increases progressively from the time of starting eculizumab treatment (a representative patient in Fig. 1a), until a relatively stable plateau is reached after 10–12 weeks: 5.8 ± 3.4% at 1 week (*n* = 8), 16.9 ± 13,3% at 4–5 weeks (*n* = 8), 20.5 ± 6.6% at 10–12 weeks (*n* = 8). In a series of 41 PNH patients on stable eculizumab treatment, we have previously observed 27.2 ± 18.7% of C3+ PNH RBCs [23]. PNH RBCs with bound C3 were stained by a moAb that recognizes a specific C3d neo-epitope [39], but not by an anti-C3b (unable to bind the C3d fragment) and an anti-iC3b (Fig. 1b); in addition, these C3 bound PNH RBCs were not stained by anti-Bb and anti-C3c antibodies (Additional file 1: Figure S1).

### Modeling in vitro the in vivo binding of C3 to PNH red blood cells

In order to investigate what happens in vivo in PNH patients on eculizumab, we have designed a novel experimental setting [31], whereby RBCs from PNH patients are exposed to the mild activation of complement that mimics the in vivo spontaneous activation of CAP known as C3 “tickover”. C3 “tickover” occurs because of the spontaneous continuous and low-rate hydrolysis of C3, followed by its interaction with Factor B, which eventually leads to the formation of the initial fluid phase C3 convertase (C3:H_2_OBb) [39–44].

When we incubated RBCs from a PNH patient in normal sera at 37 °C, we observed after 5 days a significant degree of hemolysis [36]. The ghosts arising from hemolysis were exclusively GPI-negative (ghosts of PNH RBCs), and nearly all of them bound C3 fragments (Additional file 1: Figure S2). In contrast, none of the intact RBCs have bound C3 fragments confirming that we are mimicking the continuous chronic intravascular hemolysis [32, 37] occurring in vivo in PNH patients [8, 45].

**Fig. 2:**
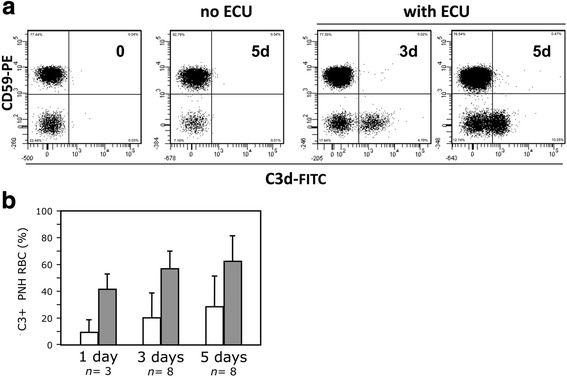
Effect of spontaneous complement activation on PNH red cells. **a** Kinetics of C3 binding on PNH red cells from a representative PNH patient not on eculizumab after spontaneous complement activation in presence of eculizumab. The *first panel* shows that no C3 binding is present on red cells from patients not on eculizumab. The *second panel* shows that there is no C3 binding on red cells after 5 days of incubation at 37 °C in ABO-compatible sera without eculizumab. C3 binding (C3+) appears on PNH (CD59-negative) red cells after 3 (*third panel*) and after 5 (*fourth panel*) days of incubation at 37 °C in ABO-compatible sera with eculizumab. The number in the *upper right angle* of each diagram indicates the time in days (d); 0d indicates the sample analyzed before any in vitro treatment. ECU: eculizumab. **b** Kinetics of C3 binding on PNH red cells from a series of PNH patients not on eculizumab after spontaneous complement activation in presence of eculizumab. The bar diagram shows the average (+SD) proportion of PNH (CD59-negative) red cells bound with C3 in a series of patients not on eculizumab after 3 and after 5 days of incubation at 37 °C in ABO-compatible sera with eculizumab. *n*: number of different patients studied at the indicated time points. *Empty bars* show the experiments without addition of MgCl_2_ whereas the *filled bars* show the experiments with the addition of MgCl_2_ (1.25 mM). C3 binding on PNH red cells increased significantly along the time (Wilcoxon signed paired test: *P <* 0.04 for the series without MgCl_2_ and *P* < 0.05 for the series with addition MgCl_2_). At both 3 and 5 days C3 binding of samples with MgCl_2_ was significantly higher than that of the same samples without MgCl_2_: *P <* 0.008, Wilcoxon signed paired test

When we repeated these experiments using sera with eculizumab, there was almost no lysis of PNH RBCs (0–10% at day 5) and we observed, already after 24 h, the appearance of a discrete population of C3+ PNH RBCs coexisting with PNH RBCs without C3 and with normal RBCs (Fig. 2a). In addition, the size of the C3+ PNH RBC population increased significantly with time (*P <* 0.04) up to 29 ± 23% after 5 days (Fig. 2b). This parallels exactly what happens in vivo in patients on eculizumab (Fig. 1a). No C3 binding was observed with sera that were previously heat-inactivated (data not shown), confirming that the phenomenon was entirely complement-mediated.

**Fig. 3:**
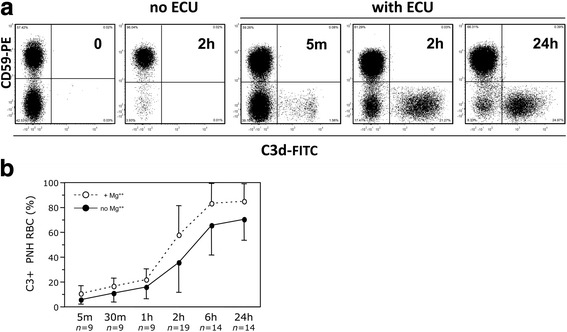
Effect of complement activation by mild acidification on PNH red cells. **a** Kinetics of C3 binding on PNH red cells from a representative untreated PNH patient after complement activation in vitro by mild acidification in presence of eculizumab. Red cells from a patient not on eculizumab incubated with acidified ABO-compatible sera without eculizumab show almost complete lysis of PNH (CD59-negative) red cells by 2 h: compare the *first panel* (0 h: before any acidification) and the *second panel* (2 h, no ECU). C3 binding (C3+) on PNH (CD59-negative) red cells is shown before (time 0) and after 5 min, 2 h and 24 h from complement activation in ABO-compatible sera with eculizumab. **b** Kinetics of C3 binding on PNH red cells from a series of PNH patients not on eculizumab after complement activation in vitro by mild acidification in presence of eculizumab. The line diagram shows the average (+SD) proportion of PNH (CD59-negative) red cells bound with C3 in a series of untreated patients at different time points (from 5 min to 24 h) since complement activation in ABO-compatible sera with eculizumab (*continuous line* without MgCl_2_; *dotted line* with 1.25 mM MgCl_2_). *n*: number of different patients studied at the indicated time points. C3 binding on PNH red cells increased significantly along the time (Friedman rank sum test: *P <* 0.001 for both series with and without MgCl_2_). At any time C3 binding of samples with MgCl_2_ was significantly higher than that of the same samples without MgCl_2_ (Wilcoxon signed paired test: *P <* 0.03 at 5 min and 1 h; *P* < 0.005 at 30 min and 2, 6, and 24 h)

Then we have carried out similar experiments in which CAP was activated by acidification (as in the Ham test) [27, 28, 32]. When RBCs from patients not on eculizumab were incubated with acidified normal sera, there was the lysis of almost all PNH RBCs after 2 h (Fig. 3a). When we used sera with eculizumab, the hemolysis of PNH RBCs was largely reduced but not completely prevented (42.6 ± 12.6% after 24 h; *n* = 11). The level of this “residual” hemolysis was only slightly and not significantly reduced by adding an excess of eculizumab (400 μg/ml) that is expected to double its final concentration: 47.1 ± 12.0 vs. 43.0 ± 10.0% (*n* = 5).

In addition, when complement was activated by acidification in the presence of eculizumab, we observed the prompt appearance of a discrete population of C3+ PNH RBCs within 5 min from serum acidification (5.9 ± 3.6%) and its size increased with time up to 71 ± 17% within 24 h (Fig. 3a, b; *P <* 0.001). Again, no C3 binding was observed with sera that were previously heat-inactivated. With the addition of 1.25 mM MgCl_2_ (which is known to optimize complement activation [46]), there was a further significant increase of C3+ PNH RBCs in both experimental settings (Figs. 2b, 3b) and also an increase of residual hemolysis (66.7 ± 14.7% after 24 h: *n* = 11).

**Fig. 4:**
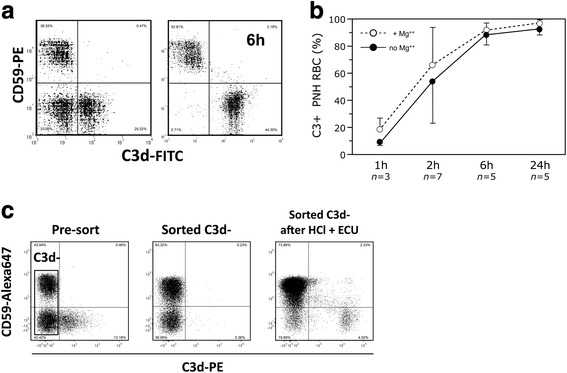
Effect of complement activation by mild acidification on PNH red cells from patients on eculizumab. **a** C3 binding on PNH red cells from a representative PNH patient on eculizumab after complement activation in vitro by mild acidification in presence of eculizumab. This experiment shows the C3 binding (C3+) on PNH (CD59-negative) red cells before (*left panel*) and after 6 h (*right panel*) from complement activation in vitro. The number in the *upper right angle* of each diagram indicates the time in hours (h); 0 h indicates the sample analyzed before complement activation in vitro. **b** Kinetics of C3 binding on PNH red cells from a series of PNH patients on eculizumab after complement activation in vitro by mild acidification in presence of eculizumab. The line diagram shows the average (+SD) proportion of C3-negative PNH red cells that became bound with C3 in a series of eculizumab-treated patients at different time points (from 1 to 24 h) since complement activation in their own sera (*continuous line* without MgCl_2_; *dotted line* with 1.25 mM MgCl_2_). In this 7 patients, the percentage PNH red cells that in vivo were already bound with C3 was 30.3 ± 24.5%. *n*: number of different patients studied at the indicated time points. **c** Effect of in vitro complement activation on C3− PNH red cells from patients on eculizumab*.* RBCs without C3 binding have been sorted by FACS from a patient on eculizumab and subjected to complement activation in the presence of eculizumab. Analysis of RBC with anti-CD59 and anti-C3d: just after sample drawing (*left panel*); after FACS sorting of C3− RBC (*middle panel*); sorted C3− RBC after 2 h from complement activation in presence of eculizumab (*right panel*). HCl: serum acidification. ECU: eculizumab

Furthermore, when RBCs from patients on eculizumab, who already had both populations of C3+ and C3− PNH RBCs, were incubated with their own acidified sera, nearly all the C3− PNH RBCs eventually became C3+ (Fig. 4a, b). This held true also for the C3− RBCs physically sorted from these patients: in fact, upon complement activation, also these sorted C3− PNH RBCs became C3+ (Fig. 4c).

### C3 binding to young (reticulocytes) and to mature PNH red blood cells

**Fig. 5:**
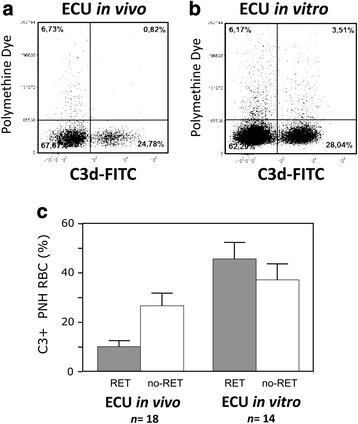
C3 binding on young (reticulocytes) and mature (non-reticulocytes) PNH red cells in the presence of eculizumab in vivo and in vitro. Analysis of PNH (CD59-negative) red cells for reticulocyte staining (polymethine dye) and C3 binding in a representative patient whose red cells has been studied both **a** in vivo (after 6 months of eculizumab treatment: *left panel*) and **b** in vitro (before starting eculizumab: *right panel*). In the in vitro experiment, red cells have been incubated for 2 h with acidified ABO-compatible sera from patients on eculizumab without MgCl_2_ addition. Serum acidification has not induced any C3 binding to non-PNH red cells neither with nor without eculizumab (data not shown). **c** The bar diagram shows the average (+SD) proportion of young (reticulocytes: RET, *filled bars*) and mature (non-reticulocytes: no-RET, *empty bars*) PNH (CD59-negative) red cells bound with C3 in patients studied during (from 3 to 36 months from the start) eculizumab treatment (on the *left*: eculizumab in vivo) and in patients not on eculizumab studied in vitro (on the *right*: eculizumab in vitro). *n*: number of different patients. ECU: eculizumab

Since in vivo not all PNH RBCs are bound with C3, one may surmise that this could be due to intrinsic difference between the PNH RBCs that become C3+ and those that remain C3−. For instance, it could be that young RBCs have some extra mechanism for complement regulation, which would inhibit C3 binding and might be lost with aging: i.e., the surface density of glycophorin A [47], CD44, CD147 [48], and CR1 [49] decreases with RBC aging. In order to test this hypothesis, we have compared the proportion of C3+ cells among young (reticulocytes) and mature (non-reticulocytes) PNH RBCs. In samples obtained ex vivo from patients on eculizumab (*n* = 18), the proportion of C3+ PNH RBCs was much lower in reticulocytes than in non-reticulocytes (10.5% vs. 27.7%: *P <* 0.0025. Fig. 5a). Reticulocytes mature into RBCs within few hours also in vitro [50]; thus, we have studied C3 binding only after complement activation by serum acidification. In apparent contrast with what we have observed in vivo in patients on eculizumab, in untreated PNH patients (*n* = 14) a similar proportion of C3+ PNH RBCs was observed among both reticulocytes and non-reticulocytes (Fig. 5b) after in vitro incubation with acidified sera with eculizumab (Fig. 5c: 45.6 vs. 37.1%).

We have obtained direct confirmation of this scenario in 6 patients, which were tested in vitro before starting eculizumab (C3+ PNH reticulocytes and non-reticulocytes were 59 ± 21% and 53 ± 25%, respectively**)**, and re-tested in vivo after 4–8 months of eculizumab treatment (C3+ PNH reticulocytes and non-reticulocytes were 8 ± 5% and 23 ± 15%, respectively). In all these experiments, C3 binding has never been observed on non-PNH (GPI-positive) reticulocytes (data not shown).

## Discussion

The binding of C3 fragments to RBCs, characteristic of various autoimmune hemolytic anemias, had never been found in PNH and it has emerged as a novel phenomenon in PNH patients on eculizumab [23, 24, 51]. It is possible to visualize that in the absence of eculizumab no C3+ RBCs are seen because, once C3 is activated on the surface of PNH RBCs, they will promptly undergo hemolysis. In contrast, when C5 is blocked by eculizumab, PNH RBCs with bound C3 survive because they are no longer lysed by the terminal complement pathway; however, there will be now a “new” steady state in which part of these C3-opsonized RBCs are removed by macrophages, likely via interaction with the complement receptor 3 [52] producing the extravascular hemolysis [23].

This “new” steady state does not detract from the clinical benefit arising from the abrogation of intravascular hemolysis. However, this observation accounts for the fact that only a minority of PNH patients on eculizumab normalizes their hemoglobin level, and in some patients, residual anemia may even require red blood cell transfusions [14, 15, 20, 23, 24]. For these patients there are not yet standard treatments: steroids have been proven to be ineffective [51]; splenectomy [53] or splenic artery embolization [54, 55] may be effective but raise concerns about infection and thrombosis susceptibility [15].

We have effectively reproduced in vitro the phenomenon of C3 binding: in fact, in vitro complement activation in the context of C5 blockade generates a distinct population of C3+ PNH RBCs which co-exists with C3− PNH RBCs. It is intriguing that spontaneous complement activation in vitro, a condition that may mimic the chronic low level of complement activation present in vivo, results in the same order of C3 binding we have previously observed in vivo in PNH patients (*n* = 41) on eculizumab: 29 vs. 27% [23].

In addition, this mild spontaneous complement activation is almost unable to lyse PNH RBCs (less than 10% after 5 days), whereas serum acidification results in a non-negligible hemolysis of PNH RBCs (~42% after 24 h) that is not prevented by an excess of eculizumab. This is because serum acidification forces a level of complement activation in excess of what happens in vivo in the steady state [37, 56] and that might be similar to the massive complement activation present during infection or inflammation. This may explain the hemolytic crisis observed in vivo in PNH patients on eculizumab in these specific clinical circumstances, providing evidence for a “pharmacodynamic breakthrough” [57]. Our observations [31, 58] are in keeping with the recent finding that strong complement activation overrides C5 inhibition by eculizumab possibly due to the generation of high density C3 products on the RBC surface [59].

In any event, the most singular feature of C3 binding in PNH patients on eculizumab is that, despite all PNH RBCs are uniformly lacking GPI-anchored proteins, there are always two distinct PNH RBC populations with or without C3 binding. This is different from other conditions, such as the paradigmatic cold agglutinin disease [60], in which C3 binding is present on all RBCs. Thus, C3 binding in PNH patients on eculizumab emerges as a unique phenomenon with surprising features.

In principle, these two distinct populations might arise from (a) their different ability to bind C3 or (b) from a stochastic effect. However, when C5 is blocked by eculizumab, there was the prompt appearance of a discrete population of C3+ PNH RBCs whose size increased not only with time but also with the level of complement activation. In fact, in vitro the proportion of C3+ PNH RBCs after mild spontaneous complement activation (Fig. 2b) was smaller than after more intense complement activation by acidification (Fig. 3b). Moreover, in vitro, at variance with in vivo observations, eventually all PNH RBCs become C3+. This holds true also for C3− PNH RBCs from patients on eculizumab that, in vitro, become all C3+, regardless of the size of C3+ PNH RBCs population in vivo (Fig. 4b). Finally, young RBCs are not selectively protected from C3 binding, in fact in vitro a very similar proportion of young (reticulocytes) and mature (non-reticulocytes) PNH RBCs become C3+ (Fig. 5b, c).

Altogether, these results indicate that the C3+ and the C3− PNH RBCs are not intrinsically different: any PNH RBCs, when exposed to complement activation in the context of C5 blockade, may bind C3 to the same extent. Thus, C3 binding is not a prerogative of a discrete subset of PNH RBCs. In addition, the prompt appearance, within 5 min from serum acidification (Fig. 3a), of two distinct populations of PNH RBCs (with and without C3) suggests that C3 binding is an *all-or-nothing* phenomenon in which a detectable level of C3 fragments stably bound to PNH RBCs is generated only when complement activation exceed a minimal threshold.

These findings support a stochastic model in which the longer each individual RBC circulates, the higher the probability to be exposed in specific districts of the bloodstream to levels of complement activation that exceeds the threshold able to trigger C3 binding: for this reason in patients on eculizumab in vivo, the percentage of C3+ cells is much lower in reticulocytes (10.5%) than in mature RBCs (27.7%).

On the other hand, this stochastic model may also suggest that complement activation, rather than be always systemic and generalized, may also occur within localized spaces and within a limited time frame. Indeed, different organs might harbor specific mechanisms of activation and regulation of complement. This finely regulated homeostasis could explain why in most complement-mediated diseases the clinical presentations and complications are often organ specific (e.g., abdominal or central nervous vein thrombosis in PNH, renal or ocular involvement in genetically determined hemolytic-uremic syndrome and age-related macular degeneration) [61].

## Conclusions

This in vitro study helps in understanding what happens in vivo in PNH patients on eculizumab and it paves the way for alternative strategies to overcome the clinical consequences of C3 binding in PNH patients who already benefit, completely or partially, from C5 blockade [34, 57]. For example, since the stochastic nature and the *all-or-nothing* characteristic of this process, it is possible to hypothesize that in the context of C5 blockade, the prevention of C3 binding would not require a complete inhibition/full blockade of C3 convertase activity. Indeed, even a partial inhibition of C3 convertase activity, just below the threshold that triggers irreversible C3 binding, might result in meaningful clinical benefit with less concerns about the possible infectious risk associated with a complete disabling of the complement pathway.

## Additional file

Additional file 1: Figure S1. In vivo C3 binding on red cells of PNH patients on eculizumab. **Figure S2.** Effect of spontaneous complement activation on PNH red cells. (PDF 1927 kb)

## Abbreviations

C3: Complement component 3
C5: Complement component 5
CAP: Complement alternative pathway
*CR1*: Complement receptor 1
GPI: Glycosylphosphatidylinositol
LDH: Lactate dehydrogenase
PNH: Paroxysmal nocturnal hemoglobinuria
RBC(s): Red blood cell(s)
