# Supplementary material for: Eculizumab treatment: stochastic occurrence of C3 binding to individual PNH erythrocytes
Source: J Hematol Oncol. 2017 Jun 19;10:126. doi: 10.1186/s13045-017-0496-x (PMC5477256; doi:10.1186/s13045-017-0496-x)
Supplement: Additional file 1: Figure S1. — In vivo C3 binding on red cells of PNH patients on eculizumab. Figure S2. Effect of spontaneous complement activation on PNH red cells. (PDF 1927 kb) [file 13045_2017_496_MOESM1_ESM.pdf]

Figure S1

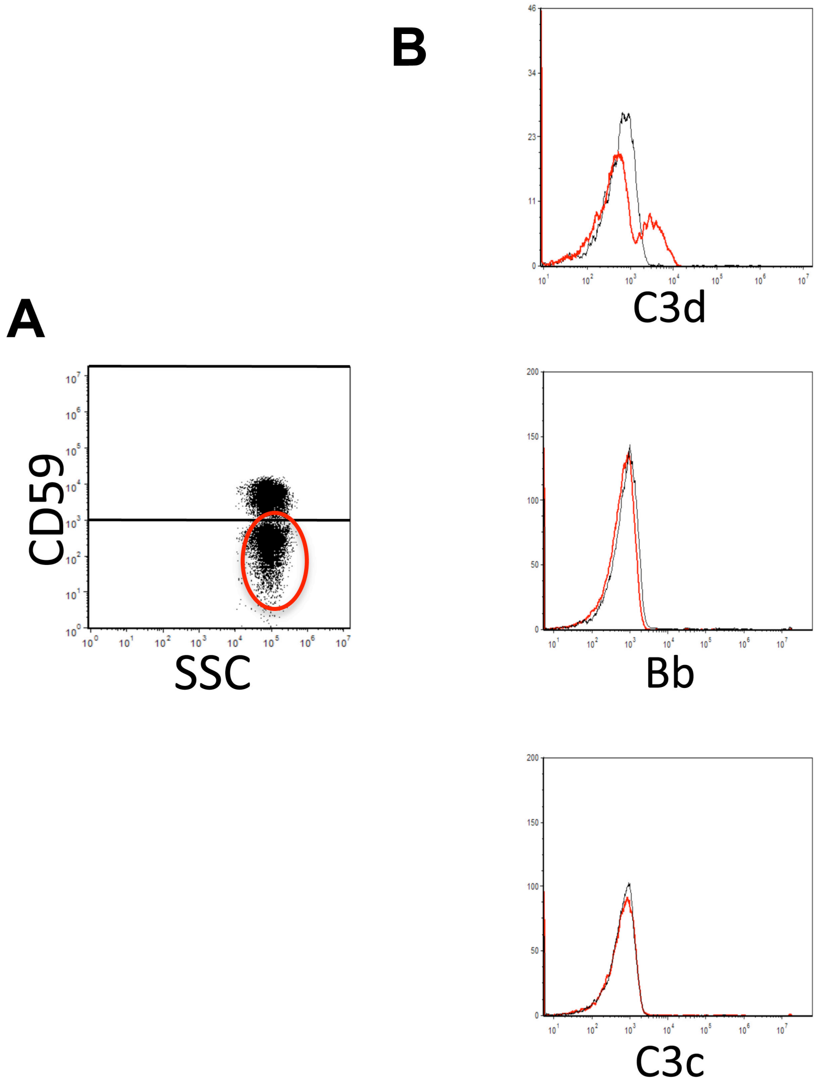

Figure S1. ***In vivo C3 binding on red cells of PNH patients on eculizumab.***

(A) Normal (CD59-positive) and PNH (CD59-negative) populations (PNH RBCs are gated by the elliptic mark).

(B) Histogram plots of PNH (CD59-negative) population gated in panel A (grey line = isotypic control): Upper histogram shows anti-C3d (red line), Middle histogram shows anti-Bb (red line) and Lower histogram anti-C3c (red line).

Figure S2

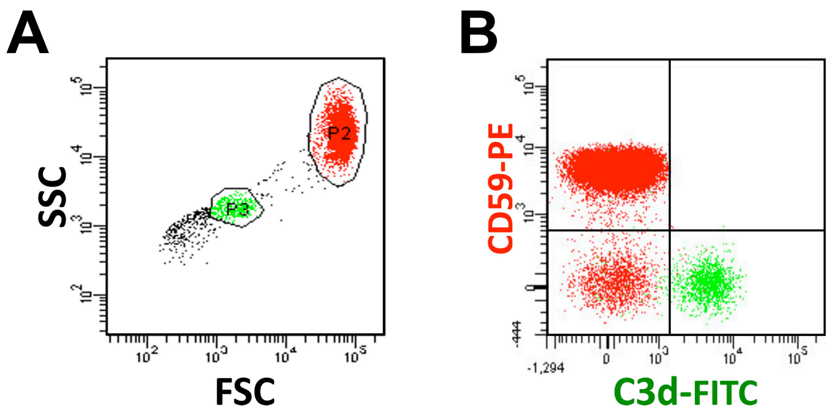

Figure S2. Effect of spontaneous complement activation on PNH red cells.

Effect of spontaneous complement activation. Red cells from PNH patients not on eculizumab are analyzed after incubation in ABO-compatible donor sera for 5 days at 37°C in sealed tube with 100% atmosphere.

(A) Ghost cells (in green) and intact red cells (in red) are identified on the basis of the physical parameters (forward –FCS- and side scatter –SSC-).[Lindorfer MA, Pawluczkowycz AW, Peek EM, Hickman K, Taylor RP, Parker CJ. Blood. 2010;115:2283-2291. DOI 10.1182/blood-2009-09-244285]

(B) Staining with anti-CD59 moAb and with anti-C3d moAb: after the spontaneous complement activation all ghost cells (in green) are PNH (CD59-negative) and almost all of them are bound with C3d fragments, whereas none of intact red cells (in red) are bound with C3d. In selected experiments the identity of intact and of ghost RBCs has been confirmed by staining with anti-Glycophorin A moAb (GA-R2, BD Becton Dickinson, NJ, USA) (data not shown).
